# Orthographic familiarity, phonological legality and number of orthographic neighbours affect the onset of ERP lexical effects

**DOI:** 10.1186/1744-9081-4-27

**Published:** 2008-07-04

**Authors:** Alice M Proverbio, Roberta Adorni

**Affiliations:** 1 University of Milano-Bicocca, Department of Psychology, Milan, Italy

## Abstract

**Background:**

It has been suggested that the variability among studies in the onset of lexical effects may be due to a series of methodological differences. In this study we investigated the role of orthographic familiarity, phonological legality and number of orthographic neighbours of words in determining the onset of word/non-word discriminative responses.

**Methods:**

ERPs were recorded from 128 sites in 16 Italian University students engaged in a lexical decision task. Stimuli were 100 words, 100 quasi-words (obtained by the replacement of a single letter), 100 pseudo-words (non-derived) and 100 illegal letter strings. All stimuli were balanced for length; words and quasi-words were also balanced for frequency of use, domain of semantic category and imageability. SwLORETA source reconstruction was performed on ERP difference waves of interest.

**Results:**

Overall, the data provided evidence that the latency of lexical effects (word/non-word discrimination) varied as a function of the number of a word's orthographic neighbours, being shorter to non-derived than to derived pseudo-words. This suggests some caveats about the use in lexical decision paradigms of quasi-words obtained by transposing or replacing only 1 or 2 letters. Our findings also showed that the left-occipito/temporal area, reflecting the activity of the left fusiform gyrus (BA37) of the temporal lobe, was affected by the visual familiarity of words, thus explaining its lexical sensitivity (word vs. non-word discrimination). The temporo-parietal area was markedly sensitive to phonological legality exhibiting a clear-cut discriminative response between illegal and legal strings as early as 250 ms of latency.

**Conclusion:**

The onset of lexical effects in a lexical decision paradigm depends on a series of factors, including orthographic familiarity, degree of global lexical activity, and phonologic legality of non-words.

## Background

Since the early 80s, one major topic of investigation has been into the exact time the brain takes to access the lexical properties and conceptual meaning of a word, after it has been presented visually or acoustically [1-3]. A lively debate has developed since then [4-6] about the timing of semantic processes, which now seem to be much earlier (150 ms) than previously conceived (about N400 ms), and to occur in parallel (rather than in sequence) with other types of speech/sentence processing (i.e. orthographic/phonological analysis, first and second order syntactic analysis, pragmatic analysis).

In addition, the ERP and MEG literature has provided conflicting evidence about the onset of lexical effects deriving from either word/non-word contrasts [3] or word expectancy and association effects [7,8], and from word familiarity [9], category/domain [10-12], word class [13], frequency of use [14] or priming [15-17] effects on the latency and amplitude of ERP/MEG components. The onset of lexical processing as described in the available literature seems to range from 110 ms [4,6] to 150 ms [18-22] up to 300/400 ms [15,17,23].

This wide variability seems to depend heavily on methodological factors [6,24] such as differences among studies in experimental parameters (e.g. word luminance, length, duration, frequency of use, semantic category or domain, grammatical class, repetition rate, familiarity, abstractness, ISI, SOA) and task modalities (lexical decision, orthographic or phonetic decision, semantic priming, SRVP, terminal word paradigm, etc.). The degree of fluency and age of acquisition of a language for a multilingual speaker [25,26], and even the number of languages known, are also very important in determining the speed of semantic processing. For example, a linear relationship has been demonstrated between response times to semantically congruent words in simultaneous interpreters engaged in a simple semantic task in their native language (judging the degree of semantic integration between a sentence and its terminal word) and the number of languages mastered by them: the response slows as the number of languages mastered increases from 3 to 5–6 [27]. Consistently, another study [28] found that the N1 and N400 components to semantically incongruous words had slower latencies in simultaneous interpreters (mastering up to 5–8 languages) than in age-matched monolingual controls. Therefore it seems that semantic processing relies on systems with limited capacity, and the speed of processing may depend on multiple factors such as those previously reported. One obvious factor in the inconsistency among studies is the inter-study variability in signal-to-noise ratio for ERP averages: in some studies, ERP waveforms are so noisy that the first reliable component showing stimulus-related effects necessarily becomes the largest in amplitude and most resistant to noise (N400), the late latency of which is thereafter considered the onset of semantic processing.

One further factor that might affect the temporal onset of the first semantic effect in lexical decision tasks based on word/non-word recognition is the orthographic similarity between words and non-words, that is the number of orthographic neighbours of pseudo-words [29,30]. Indeed, the decision processes that lead to the determination of whether a given item exists may demand more effort when a pseudo-word is orthographically quite similar to a real word. In some studies the procedure adopted to generate legal pseudo-words consists in changing one single letter in each element of a set of real words, or by transposing 1–2 letters [31]. The pseudo-words thus obtained (although meaningless) are very similar in form to words at both the orthographic and phonological levels. Interestingly, a recent ERP study [32] involving a lexical decision task (word/non-word discrimination) demonstrated that responses to pseudo-words that were perceptually similar to words, obtained by transposing two letters, were 118 ms slower than responses to less word-like pseudo-words (created by replacing those two letters). Furthermore, the transposed-letter pseudo-words activated their corresponding base words to a considerable degree, as shown by a substantial false alarm rate. As for the ERP data, the N400 component (300–500 ms) was larger to less "word-like" stimuli than to transposed-letter pseudo-words, which were treated almost as words, whereas in a second latency range (500–680 ms) this effect was reversed – transposed-letter pseudo-words were fully recognized as meaningless.

It has been shown [30] that reaction times to non-words are longer when these stimuli have many word neighbours. According to Grainger and Jacobs, non-words with many neighbours (some of which are words) generate high levels of global lexical activity through the activation of word neighbour representations. This high global lexical activity prolongs the processing time needed to determine the level of semantic denotation of a string and therefore results in slower correct 'no' responses to non-words with many neighbours. It has been consistently shown [33] that, when the pseudo-words are created by replacing one internal letter of a base word, high-frequency pseudo-words yield slower latencies than low-frequency pseudo-words in lexical decision tasks.

Braun and colleagues [34] recently investigated the role of non-word orthographic neighbours by comparing ERP responses to 300 words and 300 non-words obtained by replacing 1, 2, 3 or 4 letters from a set of 3000 real ones. They expected a systematically graded variation in the ERP, in particular of the N400 amplitude, in response to non-words. The results from a lexical decision task provide evidence for an overall effect of lexicality (word vs. pseudo-word distinction between 300 and 390 ms, and a graded effect of global lexical activity for non-words between 450 and 550 ms post-stimulus). The data are interpreted as reflecting two different decision processes: an identification process based on local lexical activity underlying the 'yes' response to words, and a temporal deadline process underlying the 'no' response to non-words based on global lexical activity.

As for the acoustic phonetic modality, an interesting ERP study [35] presented spoken words and pseudo-word variants that differed only in their medial consonants. For each pseudo-word, one phoneme was replaced with a new one, which either had a coronal (dental or nasal /d/, /t/, /n/) or a non-coronal (labial: /b/, /p/, /m/; dorsal /g/, /k/) place of occlusion. ERPs were not time-locked to stimulus onset but to deviation points. They found a marked difference in the latency of lexical effects according to the type of replacing phoneme (coronal or non-coronal). In particular, while ERPs for non-coronal variants did not differ from their base words in the initial part of the N400 (100–250 ms), the mean amplitudes for coronal pseudo-word variants were more negative than the mean amplitudes for their non-coronal base words, thus showing an early lexical effect.

The aim of the present study was to investigate further the neural mechanism subserving reading and the time course of lexical processing by comparing the bioelectrical activities elicited by letter strings with various degrees of semantic denotation (inducing a graded level of global lexical activity) and orthographic legality. For this purpose, 400 words, quasi-words (non-words with many neighbours obtained by replacing one letter), non-derived pseudo-words (non-words with few orthographic neighbours) and illegal letter strings were presented. We expected to find: (i) an effect of orthographic legality and word visual familiarity by comparing ERPs to legal pseudo-words and to illegal letter strings; (ii) a graded effect of non-word orthographic neighbours on the amplitude and latency of ERP responses, thus shedding some light on the timing of lexical processes.

## Methods

### Participants

Sixteen Italian University students (8 men and 8 women) volunteered for the study. Their ages ranged from 20 to 25 years (mean = 23; SD = 1.73). All had good or corrected-to-normal vision and right hand and ocular dominance, as attested by the Italian version of the Oldfield inventory [36]. They were all healthy and reported that they had never suffered from neurological or psychiatric diseases. Experiments were conducted with the understanding and the written consent of each participant and in accordance with ethical standards (Helsinki, 1964). The subjects earned academic credits for their participation. Four participants were excluded from the statistical analyses because of excessive EEG and EOG artefacts.

### Procedure

Stimuli consisted of 400 letter-strings including 100 Italian words, 100 legal derived pseudo-words, 100 non-derived pseudo-words, and 100 illegal letter strings. They were blue on a white background, typed in capital letters and Times New Roman font.

Derived pseudo-words were obtained by changing one single letter in an existing lemma (e.g. Banana -> Barana), whereas non-derived pseudo-words were created de novo and had no orthographic neighbours (see Table 1).

**Table 1:** Some exemplars of stimuli listed as a function of stimulus type and length.

**Length**	**WORDS**	**QUASI-WORDS**	**PSEUDO-WORDS**	**LETTER-STRINGS**
4	MORA	URSO	RALI	HRET
5	GATTO	NEPRE	IGAPA	FPCOT
6	PIOVRA	PINORO	TARGIO	RGPBLO
7	PRIMULA	STRURZO	AQUIRDA	LUAOBGD
8	USIGNOLO	GIRTILLO	FEISCOMA	AETPFITD

**Figure 1:**
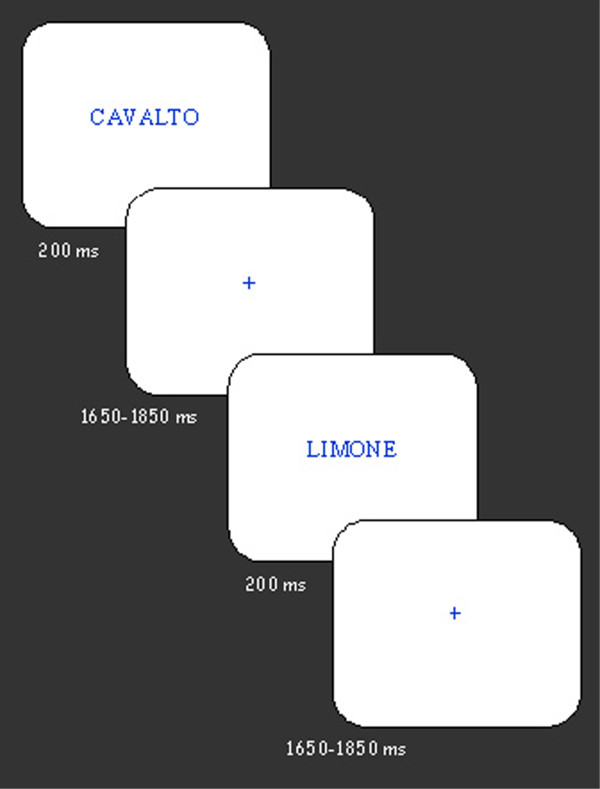
Illustration of experimental procedure, with indication of inter-stimulus interval and stimulus duration (in ms).

Stimuli were randomly presented at the central visual field for 200 ms with an ISI varying between 1650 and 1850 ms (see Figure 1). Stimuli were 1 cm in height (30'10" of visual angle) and their length ranged from 4 to 9 cm (from 2°1'41" to 4°32'32").

They were balanced for length, ranging from 4 to 8 letters (words = 6.08; SD = 1.38; pseudo-words = 6.15; DS = 1.34; quasi-words = 6.15; SD = 1.35; letter strings = 6.12; SD = 1.36). Overall, words and quasi-words (that is, the original lemmas used to generate them) were familiar and had good imageability values (half were names of animals and the other half of vegetables). Letter strings included both vocals (V) and consonants (C). The relative proportion of vocals and consonants was similar across lexical classes (e.g., 3V, 4C for a 7 letter word). The repetitive insertion of consonants not very frequent in the Italian orthography (e.g., Q, Z, X, Y, W) was also avoided. Apart from that, LS were unpronounceable and illegal, for example they did not always end in a vowel, as instead required by Italian orthographic rules.

Words and quasi-words (that is, the original lemmas used to generate them) were balanced in frequency of use according to a online database [37]. In detail, words had a mean frequency value of 22.11 (SD = 33.67); words used to generate quasi-words had a mean frequency value of 20.51 (SD = 34.31); again, for quasi-words, half were names derived from animals and the other half from vegetables. Words, quasi-words and pseudo-words were regularly pronounceable, whereas letter strings were phonologically illegal.

Participants sat comfortably in a darkened, acoustically and electrically shielded box in front of a computer screen located 114 cm from their eyes. They were instructed to fixate a little cross located at the centre of the screen and avoid any eye or body movements during the recording session.

The task was a lexical decision task (word/non-word). Subjects had to press a button with the index finger (of the left or right hand) in response to words, and with the middle finger in response to non-words, as accurately and rapidly as possible. The two hands were used alternately during the recording session, and the hand and sequence order were counterbalanced across subjects.

### EEG recording and analysis

**Figure 2:**
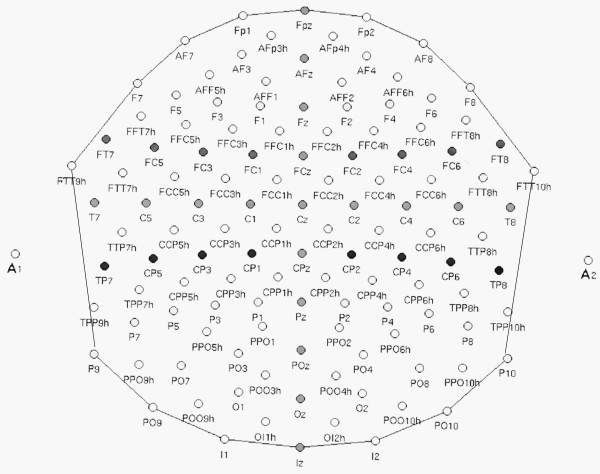
Scheme of the 128 channels electrode montage.

The EEG was continuously recorded from 128 scalp sites (see Figure 2 for the complete electrode montage) at a sampling rate of 512 Hz. Horizontal and vertical eye movements were also recorded. Linked ears served as the reference lead. The EEG and electro-oculogram (EOG) were amplified with a half-amplitude band pass of 0.016–100 Hz. Electrode impedance was kept below 5 kΩ. EEG epochs were synchronized with the onset of stimulus presentation and analyzed using ANT-*EEProbe *software. Computerized artefact rejection was performed before averaging to discard epochs in which eye movements, blinks, excessive muscle potentials or amplifier blocking occurred. EEG epochs associated with an incorrect behavioural response were also excluded. The artefact rejection criterion was a peak-to-peak amplitude exceeding 50 μV, and the rejection rate was ~5%. ERPs were averaged off-line from -100 ms before to 1000 ms after stimulus onset.

Response times exceeding mean ± 2 standard deviations were excluded. Hit and miss percentages were also collected and arc sin transformed in order to be statistically analyzed. Behavioural (both response speed and accuracy data) and ERP data were subjected to multifactorial repeated-measures ANOVA. The factors were "lexical class" (words, quasi-words, pseudo-words, letter strings) and "response hand" (left, right) for RT data, and additionally "electrode" (dependent on ERP component of interest) and "hemisphere" (left, right) for ERP data. Multiple comparisons of means were done by post-hoc Tukey tests.

Topographical voltage maps of ERPs were made by plotting colour-coded isopotentials obtained by interpolating voltage values between scalp electrodes at specific latencies. *Low Resolution Electromagnetic Tomography *(LORETA [38] was performed on ERP difference waves at various time latencies using *ASA3 *and *ASA4 *software. LORETA, which is a discrete linear solution to the inverse EEG problem, corresponds to the 3D distribution of neuronal electric activity that has maximum similarity (i.e. maximum synchronization), in terms of orientation and strength, between neighbouring neuronal populations (represented by adjacent voxels). In this study an improved version of Standardized Low-Resolution brain Electromagnetic Tomography (sLORETA) was used that incorporates a singular value decomposition-based lead field weighting: swLORETA [38,39]. Source space properties were: grid spacing = 5 mm; Tikhonov regularization: estimated SNR = 3.

ERPs were analyzed by considering three factors for variance: "lexical class" (words, quasi-words, pseudo-words, and letter strings), "electrode" (depending on the component of interest), "hemisphere" (left, right).

The mean amplitude of temporal P2/N3 and P3 components was measured at centro-parietal (CP5, CP6) and temporo/parietal (TTP7, TTP8h) sites between 250 and 350 ms, and between 380 and 460 ms, respectively. The mean amplitude of occipito/temporal N3 was measured at lateral occipital (PO9, PO10) and posterior temporal sites (P9, P10) between 345 and 395 ms. The mean amplitude of N400 response was measured at the same sites between 400 and 600 ms. This ANOVA was performed on ERP responses to legal strings (words, quasi-words, pseudo-words).

P3 peak latency and peak amplitude were measured at CP5, CP6 sites between 380 and 730 ms post-stimulus. Measurements in the ascending phase of P3 component (mean amplitude value in the 380–460 ms time window) were performed to emphasize the quite early P3 response to letter strings.

In order to focus the analyses on the mechanisms supporting lexical processing and to explore the graded effect of global lexical activity for the three categories of legal strings, further ANOVAs were performed on anterior components, with three levels of variability for "lexical class factor" (words, quasi-words, pseudo-words). Anterior and central components were measured as follows: N2 mean amplitude between 200 and 250 ms at the FFC1h, FFC2h, FFC3h, FFC4h electrode sites. Late negative deflection *lexical processing negativity *(LPN) mean amplitude was measured between 250 and 340 ms at the AFF1, AFF2, AFp3h, AFp4h electrode sites. This components has been described by King and Kutas [14] as an anterior negativity, ranging from about 280 to 385 ms of latency, and being very sensitive to the frequency of occurrence of words.

P3 component mean amplitude was measured between 340 and 400 ms at the AFF1, AFF2, AFp3h, AFp4h electrode sites. P/N400 mean amplitude was measured between 400 and 600 ms at the CCP5h, CCP6h, CPP5h, CPP6h sites whereas P600 mean amplitude was measured between 600 and 800 ms at the same electrode sites.

## Results

### Behavioural data

ANOVA performed on accuracy data (incorrect categorizations) revealed the significance of lexical class (F3,33 = 21.584; p < 0.001; eta2 = 0.662; F-crit = 2.89), showing a higher error percentage to words and quasi-words, incorrectly judged as meaningless and meaningful, respectively, (W = 6.69 %; QW = 4.42 %) than to pseudo-words and letter-strings, (PS = 1.07 %; LS = 0.51 %), as proofed by post-hoc comparisons (p < 0.001). Omissions were very few (W = 0.35%; QW = 0.525%; PS = 0.525%; LS = 0.875%) and they did not statistically differ across lexical classes, as shown by an ANOVA performed on misses percentages.

ANOVA on the RTs revealed the effect of lexical class (F3,33 = 8.0639; p < 0.001; eta2 = 0.423; F-crit = 2.891), showing that RTs were most rapid to letter strings and slowest to quasi-words (W = 554; QW = 622; PS = 567; LS = 532 ms). Post-hoc comparisons showed that response times were slower in response to quasi-words than to any other stimulus type (p < 0.01), while they tended to be faster to letter strings than pseudo-words (p = 0.07), probably reflecting task difficulty. Response hand had no effect on behavioural data.

### Electrophysiological data

#### Posterior components

##### Occipito/temporal N3 (345–395 ms)

**Figure 3:**
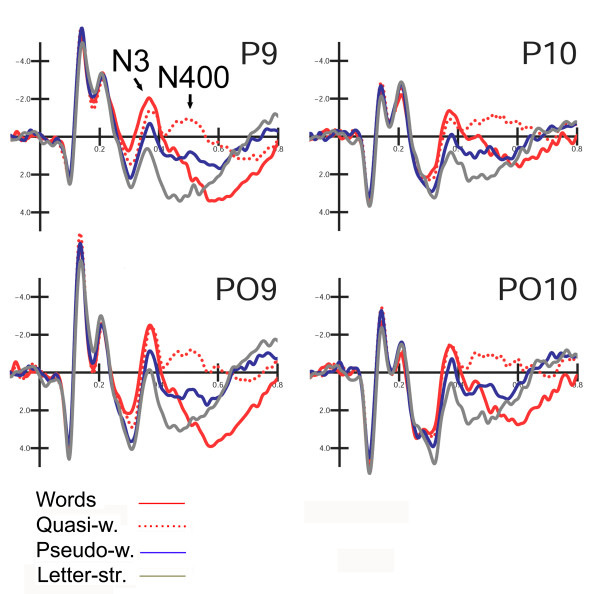
Grand-average ERP waveforms recorded at left and right ventral lateral occipital (P9, P10) and occipito/temporal (PO9, PO10) sites in response to words, derived non-words (Quasi-w.), pseudo-words (Pseudo-w.) and letter strings (Letter-str.).

Figure 3 shows the grand-average ERP waveforms recorded at posterior sites in response to the various stimulus types. N3 was strongly affected by lexical class (F3,33 = 13.99; p < 0.001; eta2 = 0.56; F-crit = 2.891) showing a gradient of activation with larger amplitudes for words and smaller for letter strings (W = -1.45; QW = -0.99; PS = -0.27; LS = 0.93 μV). Post-hoc comparisons indicated a significant difference between words and pseudo-words (p < 0.05), no difference between words and quasi-words, and a marked difference between legal (words, quasi-words and pseudo-words) and illegal strings (p < 0.001).

**Figure 4:**
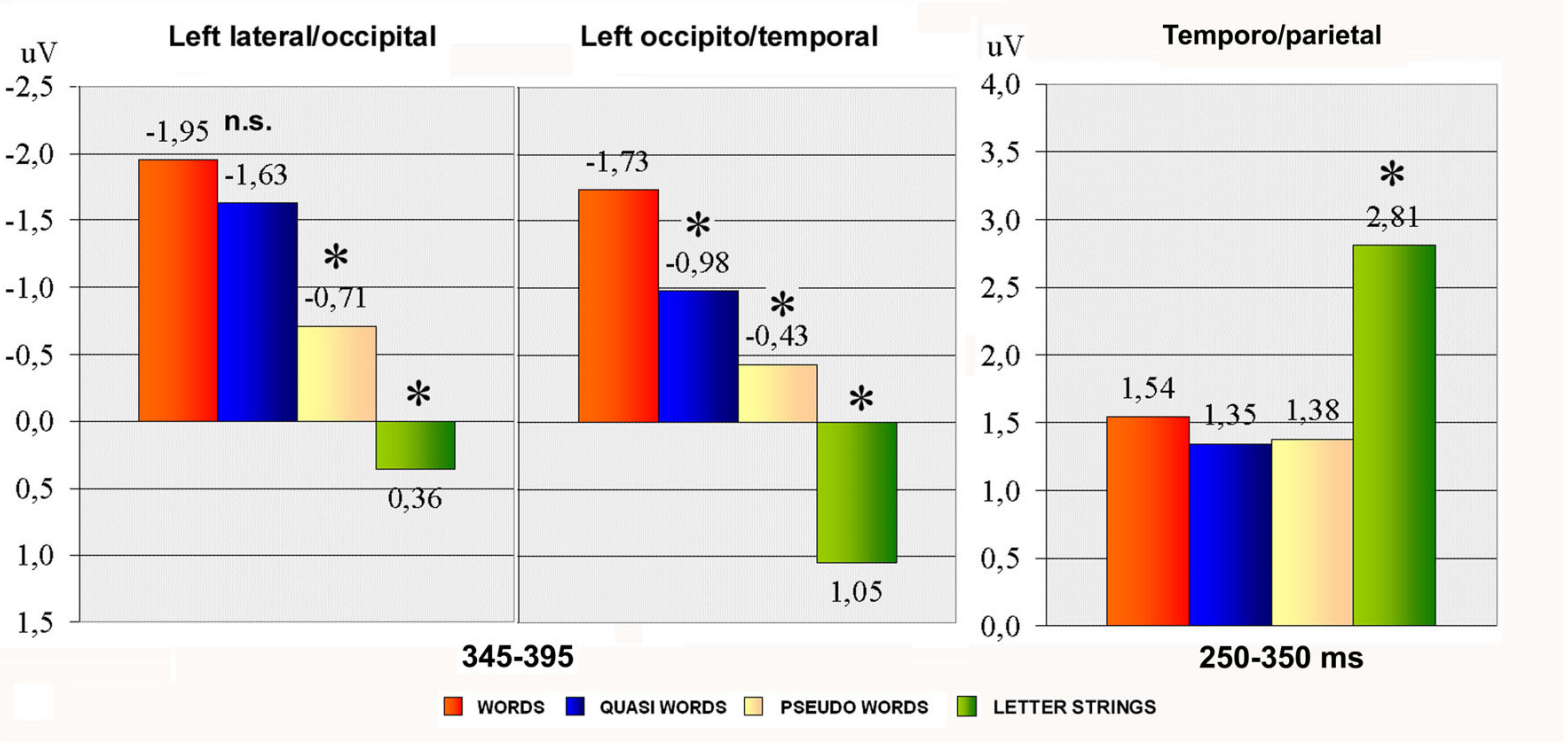
**Summary of well-formedness and string legality effects for the amplitude of left lateral-occipital N3 (LEFT), occipito/temporal N3 (MIDDLE) and temporo/parietal P2/N3 responses. **Mean amplitude values were recorded between 345 and 395, and 250 and 350, respectively.

The interaction lexical class × electrode × hemisphere (F3,33 = 3.71; p < 0.021; eta2 = 0.252; F-crit = 2.891) showed larger lexical effects at left than at right electrode sites, and a significant difference between N3 to words and quasi-words at the occipito/temporal (p < 0.001) but not the lateral occipital site. Overall, the effects of orthographical well-formedness and legality were larger at the former than the latter, as illustrated by the mean N3 values plotted in Figure 4.

##### N400 response (400–600 ms)

The significance of "lexical category" (F2,22= 11.75; p < 0.001; eta2 = 0.516; F-crit = 3.443) indicated a much larger N4 to quasi-words than pseudo-words (see waveforms in Figure 3). Post-hoc comparisons indicated a significant difference (p < 0.01) between quasi-words and pseudo-words with larger N400's to QW (-0.53 μV) than PS (0.97 μV), and between words and pseudo-words, with larger responses to PS than W (1.51 μV; p < 0.001).

The interactions "Lexical category × Electrode" (F2,22= 12.69; p < 0.001; eta2 = 0.535; F-crit = 3.443) and "Lexical category × Hemisphere" (F2,22= 4.63; p < 0.02; eta2 = 0.296; F-crit = 3.443) showed that differences according to word type were more evident at lateral occipital than at occipito/temporal sites (PO9–PO10: W = 1.85; QW = -0.58; PS = 1.00 μV; P9–P10: W = 1.18; QW = -0.49; PS = 0.93 μV), and over the left than the right hemisphere (LH: W = 1.99; QW = -0.41; PS = 1.04 μV; RH: W = 1.04; QW = -0.66; PS = 0.89 μV).

#### Centro/parietal components

##### P2/N3 (250–350 ms)

**Figure 5:**
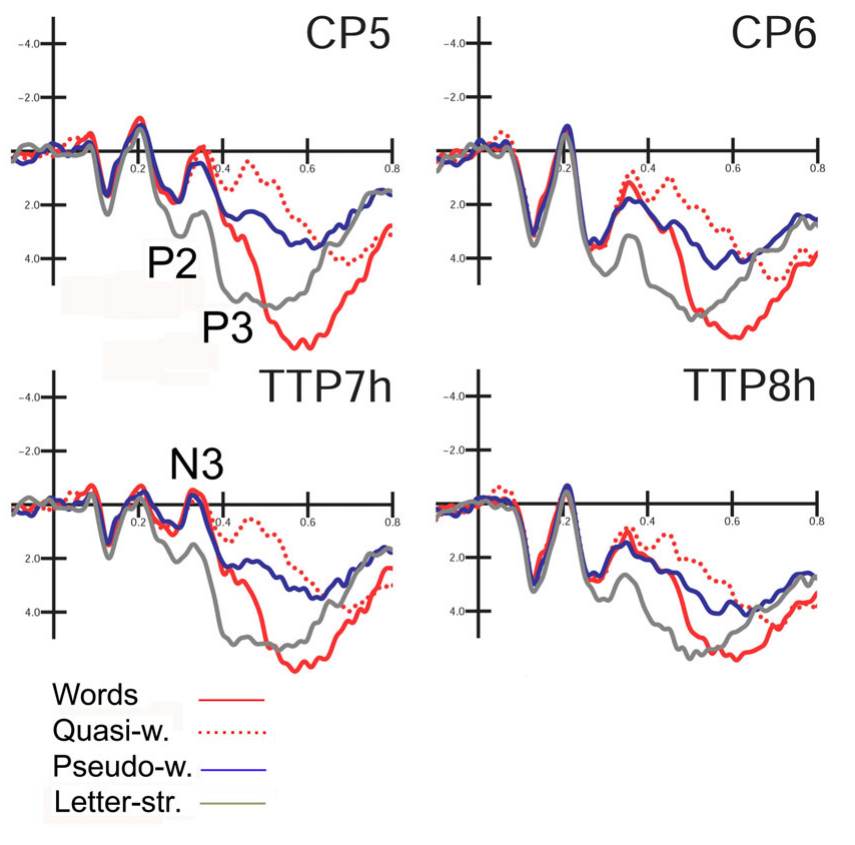
Grand-average ERP waveforms recorded at temporo/parietal and centro-parietal sites in response to the various stimulus types.

Figure 5 shows grand-average ERP waveforms recorded at the temporo/parietal and centro-parietal sites in response to the various stimulus types. The effect of orthographic legality was much earlier at this scalp region, as shown by the significance of lexical class (F3,33 = 6.7; p < 0.001; eta2 = 0.378; F-crit = 2.891); P2 was greater to illegal strings (p < 0.005) than to any other stimulus type (see Figure 4 for amplitude mean values). The electrode factor (F1,11 = 14.78; p < 0.002; eta2 = 0.573; F-crit = 4.844) yielded a larger P2 at the centro-parietal than the temporal area (CP5–CP6 = 2.14; TTP7h–TTP8h = 1.40 μV), while the hemisphere factor (F1,11 = 16.23; p < 0.002; eta2 = 0.596; F-crit = 4.844) showed a larger P2 over the right than the left hemisphere (LH = 1.01; RH = 2.53 μV).

##### P300 (380–460 ms)

At this time window, P3 amplitude was strongly affected by lexical class (F3,33 = 14.98; p <0.001; eta2 = 0.577; F-crit = 2.891), showing a much larger component to illegal strings than to any other stimulus type (W 2.44; QW = 1.10; PS = 1.92; LS = 4.75 μV). The interaction of lexical class with electrode (F3,33 = 4.36; p < 0.01; eta2 = 0.284; F-crit = 2.891) showed a more prominent P3 overall to letter strings at centro-parietal sites. Furthermore, P3 showed a gradient, probably reflecting task difficulty and decision-making processes, with larger P3 to words than pseudo-words (p < 0.001), and to pseudo-words than quasi-words (p < 0.001), at both sites.

##### P3 peak latency

The latency of the late positive component (P3) was strongly modulated by lexical class (F3,33 = 37.8; p < 0.001; eta2 = 0.774; F-crit = 2.891). Post-hoc comparisons showed shorter latencies in response to letter strings (484 ms) than to words (570 ms) or pseudo-words (588 ms; p < 0.001), and to the former than quasi-words (680 ms; p < 0.001), thus perfectly recalling the gradient shown by behavioural data.

##### P3 peak amplitude

The peak amplitude of P3 was affected by lexical class (F3,33 = 10.5; p < 0.001; eta2 = 0.488; F-crit = 2.891), showing greater responses to words (8.5 μV) than non-words, with no distinction between quasi- and pseudo-words (QW = 5.3; PW = 5.2 μV). Letter strings elicited an intermediate response (7.24 μV), probably still reflecting task difficulty. The lexical effect, and in particular the word/non-word distinction, was larger over the left hemisphere, as shown by the lexical class × hemisphere interaction (F3,33 = 2.91; p < 0.05; eta2 = 0.21; F-crit = 2.891).

#### Anterior components

##### N2 (200–250 ms)

**Figure 6:**
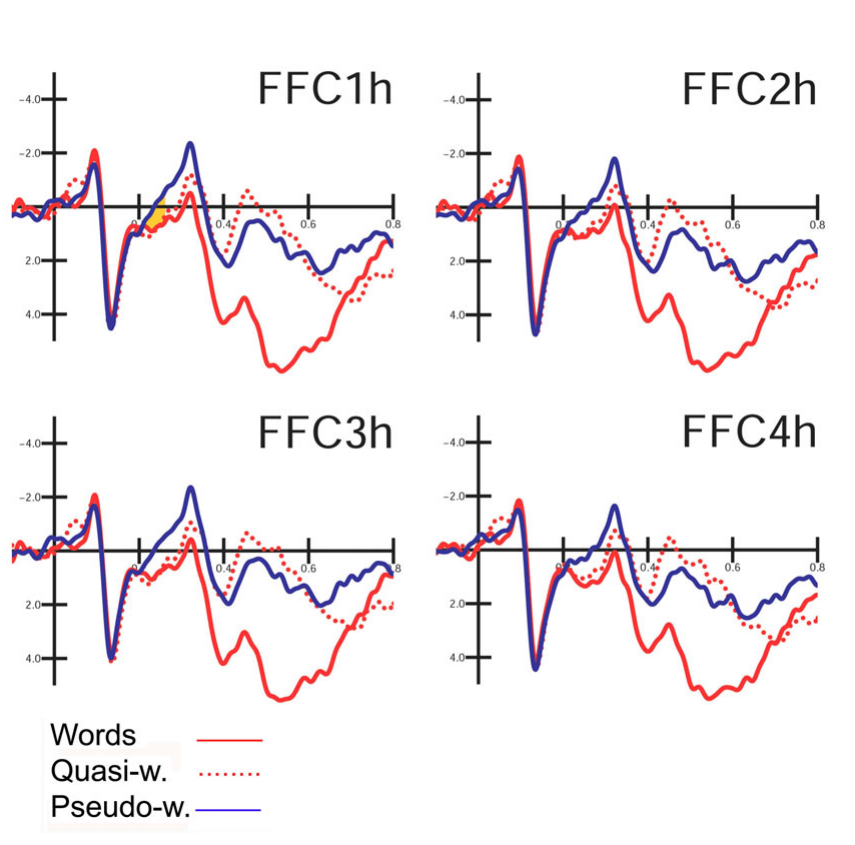
**Grand-average ERP waveforms recorded at left and right fronto-central mesial and lateral sites in response to the various stimulus types.** The early clear-cut distinction between non-derived pseudo-words and word-like stimuli (words and quasi-words) between 200 and 250 ms in the ascending early phase of LPN is visible.

**Figure 7:**
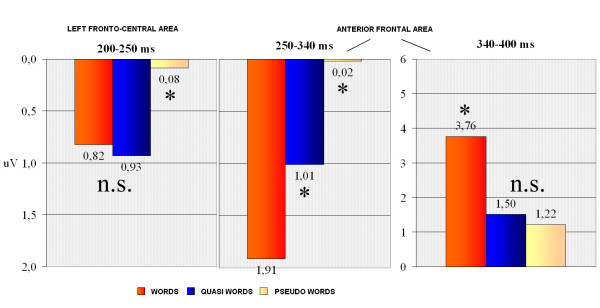
**Summary of the lexical effects for the amplitudes of the anterior N2, LPN and P3 components.** (LEFT) Amplitude values recorded between 200 and 250 ms over the left frontal area. (MIDDLE) LPN amplitude values recorded between 250 and 340 ms over the anterior frontal area (bilaterally). (RIGHT) P3 amplitude values recorded between 340 and 400 ms over the anterior frontal area.

Figure 6 shows grand-average ERP waveforms recorded at fronto-central sites in response to the various stimulus types. In the first temporal window considered, corresponding to the rising phase of anterior N2, the significant "lexical category × hemisphere" interaction (F2,22 = 5.39; p < 0.012; eta2 = 0.329; F-crit = 3.443) showed a larger negative response to pseudo-words than to words or quasi-words, with no difference between the two former classes of stimuli. The lexical effect was more consistent over the left hemisphere (LH: W = 0.82; QW = 0.93; PS = 0.08 μV; RH: W = 0.98; QW = 0.84; PS = 0.33 μV). This early negativity was larger at more medial (FFC1h–FFC2h = 0.57 μV) than lateral sites (FFC3h–FFC4h = 0.76 μV), as shown by electrode factor (F1,11 = 11.89; p < 0.005; eta2 = 0.519; F-crit = 4.844). Figure 7 shows a comparison of lexical effects as a function of the time-course of processing.

##### LPN (Lexical Processing Negativity) 250–340 ms

At a later stage, and at more anterior regions, the lexical effect (F2,22 = 19.62; p < 0.001; eta2 = 0.64; F-crit = 3.443) showed a finer gradient of activation: pseudo-words elicited the largest response (0.02 μV), quasi-words followed with an intermediate potential (1.01 μV), and words elicited the most positive potential (1.91 μV). All comparisons among means were significant. The electrode factor (F1,11 = 15.01; p < 0.0025; eta2 = 0.577; F-crit = 4.844) indicated a more anterior surface distribution for LPN deflection (AFF1–AFF2 = 0.78; AFp3h–AFp4h = 1.18 μV).

##### P300 component (340–400 ms)

**Figure 8:**
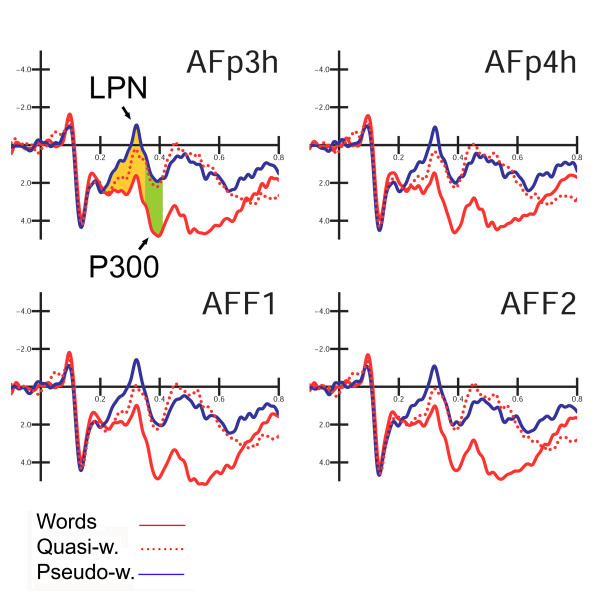
**Grand-average ERP waveforms recorded at left and right anterior frontal (AFp3h, AFp4h) and pre-frontal (AFF1, AFF2) sites in response to the various stimulus types. **A graded lexical effect for LPN component is notable, depending on the density of orthographic neighbours of the stimulus, and there is a later clear-cut discriminative effect between words and non-words.

Figure 8 shows grand-average ERP waveforms recorded at anterior-frontal sites in response to the various stimulus types. In this latency range the lexical category factor (F2,22 = 26.23; p < 0.001; eta2 = 0.705; F-crit = 3.443) was quite significant and indicated a clear difference between meaningful (W = 3.76 μV) and meaningless (QW = 1.50; PS = 1.22 μV) words, with no difference between the two categories of legal non-words, as illustrated in the graphics of Figure 7. The interaction "lexical type × hemisphere" (F2,22 = 5.316; p < 0.0013; eta2 = 0.326; F-crit = 3.443) proved a left-sided asymmetry for this effect (LH: W = 3.88; QW = 1.53; PS = 1.17 μV; RH: W = 3.63; QW = 1.48; PS = 1.26 μV).

### Late latency potentials

#### P/N400 (400–600 ms)

**Figure 9:**
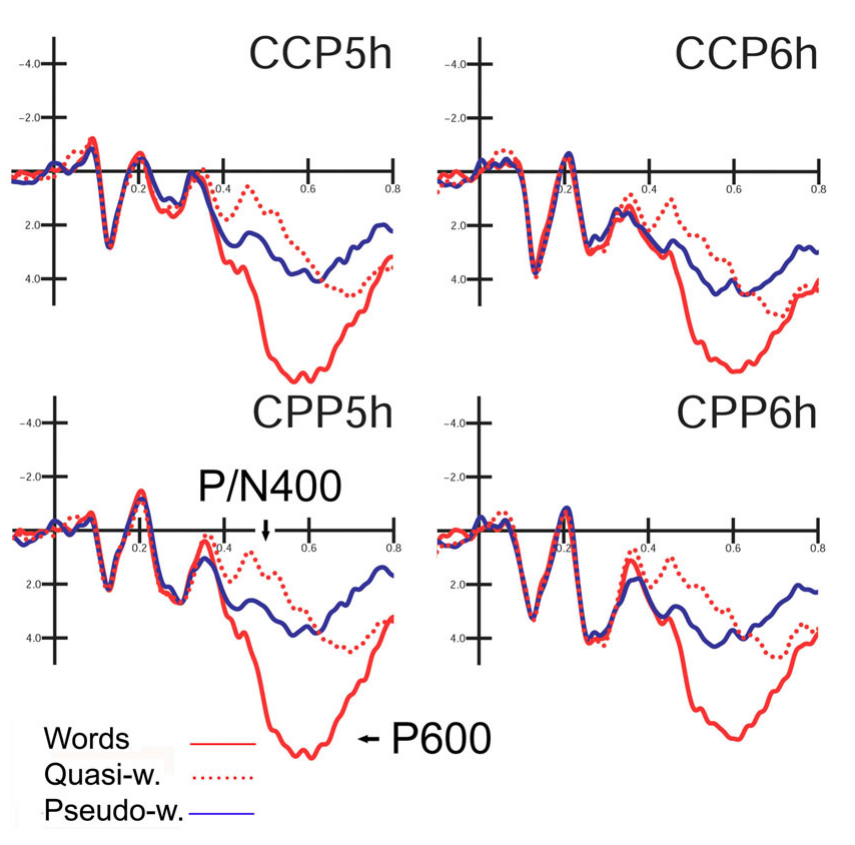
**Grand-average ERP waveforms recorded at left and right anterior and posterior centro-parietal sites in response to the various stimulus types.** The arrows indicate the larger N400 response to derived quasi-words, probably suggesting a violation of subjective expectancy.

Figure 9 shows grand-average ERP waveforms recorded at centro-parietal sites in response to the various stimulus types. In this time window, the lexical factor (F2,22 = 24.98; p < 0.001; eta2 = 0.69; F-crit = 3.443) showed a larger negativity to quasi-words than pseudo-words, and a larger positivity to words than pseudo-words (W = 5.52; QW = 1.70; PS = 2.96 μV), thus suggesting that this centro-parietal component is sensitive to subjective expectancy and semantic violation.

The interactions "lexical category × electrode" (F2,22 = 3.69; p < 0.04; eta2 = 0.25; F-crit = 3.443) and "lexical category × hemisphere" (F2,22 = 9.65; p < 0.001; eta2 = 0.467; F-crit = 3.443), showed that this effect was larger more posteriorly over the centro-parietal region (CCP5h–CCP6h: W = 5.38; QW = 1.74; PS = 2.93 μV; CPP5h–CPP6h: W = 5.66; QW = 1.66; PS = 2.99 μV), and over the left than the right hemisphere (LH: W = 5.87; QW = 1.59; PS = 2.81 μV; RH: W = 5.17; QW = 1.81; PS = 3.11 μV).

#### P600 (600–800 ms)

The lexical category factor (F2,22 = 14.53; p < 0.001; eta2 = 0.569; F-crit = 3.443) showed the existence of a gradient of activation for which words (W = 5.74 μV) elicited the largest response (p < 0.001), followed by quasi-words (3.85 μV); pseudo-words (2.72 μV) gave the smallest response.

The interaction "lexical category" × electrode" (F2,22 = 39.4; p < 0.001; eta2 = 0.782; F-crit = 3.443), also showed a more posterior distribution of the effect for this component (CCP5h–CCP6h: W = 5.65; QP = 4.06; PS = 3.04 μV; CPP5h–CPP6h: W = 5.83; QP = 3.64; PS = 2.40 μV). At both electrode sites, P600 was larger to words than to either type of non-word (p < 0.001), and to quasi-words than pseudo-words (p < 0.001), as shown by post-hoc comparisons.

## Discussion

### The role of orthographical well-formedness and visual familiarity in reading

Overall, it seems that while the left occipito/temporal area is sensitive to word visual familiarity, the temporo/parietal area is more sensitive to phonological legality. This anatomical and functional dissociation was reflected by the following. (1) There was a lack of discriminatory N3 response between real words and quasi-words, depending on their global visual resemblance to words at left occipital area. This finding suggests the existence of a visual input lexicon, which would store the visual form of known words, allow direct access to the lexicon through a visual route and show early effects of word familiarity (e.g. [6,21]). According to the dual route model of reading, damage to it would result in reading disorders such as so-called surface dyslexia [40]. (2) The ERP data also showed a gradient of lexical activation for N3 at the left occipito/temporal site in response to words with different numbers of orthographic neighbours. This finding is consistent with recent data supporting the evidence that VWFA, besides being strongly sensitive to orthographic stimulus properties [41-45], might be also sensitive to word frequency [46]. (3) At superior temporal sites, ERP showed a clear-cut discriminative response between legal and illegal strings, which was insensitive to the lexical content, probably suggesting difficulty in accessing the phonological forms of illegal strings. It might be suggested that this surface potential corresponds to intracranial generators responsible for the fast mapping between orthographic and phonological representations.

In order to locate the possible neural source of this effect, a swLORETA source reconstruction was performed on the difference-wave obtained by subtracting ERPs to pseudo-words from those elicited by letter-strings in the time window corresponding to the temporo/parietal P2/N3 (300–350 ms). The inverse solution showed that the processing of phonologically illegal strings was significantly associated with stronger activity in a series of left and right hemispheric regions, listed in Table 2, including the left angular gyrus (BA 39) and the left pre-central and post-central area. As well known, the angular gyrus is thought to play a crucial a role in phonological processing [47] and especially in grapheme to phoneme conversion [48,49]. In this context, it is possible that the so called 'dorsal phonological area', including the suvramarginal gyrus (BA 40), might become more active during reading of hardly readable material such as illegal letter strings.

**Table 2:** Letter-strings – Pseudo-words.

**Magn.**	**T-x (mm)**	**T-y (mm)**	**T-z (mm)**	**Hem.**	**Lobe**	**Area**	**BA**
1.24	-18.5	-16.1	-22.2	L	Limbic	Parahippocampal Gyrus	28
1.19	21.2	-24.5	-15.5	R	Limbic	Parahippocampal Gyrus	35
1.05	50.8	-0.6	-28.2	R	Temporal	Middle Temporal Gyrus	21
8.89	40.9	-76.2	-11.7	R	Occipital	Fusiform Gyrus	19
9.53	1.5	-20.3	26.8	R	Limbic	Cingulate Gyrus	23
6.32	-28.5	-60.8	32.3	L	Parietal	Angular Gyrus	39
6.24	-38.5	2.4	29.4	L	Frontal	Precentral Gyrus	6
5.89	-38.5	-21	35.7	L	Parietal	Postcentral Gyrus	3

Tailarach coordinates corresponding to the intracranial generators explaining the difference voltage Letter-strings – pseudo-words in the 300–350 ms time window, according to swLORETA (ASA) [38,39]; grid spacing = 5 mm; power = 37.5 μV).

The P3 amplitude reflected a much faster identification of non-words when they were also ill-formed and illegal. The lexical effect resulted in a larger P3 component to words than non-words. The smaller and later P3 to quasi-words than to pseudo-words probably reflected the difficulty of rejecting as non-words items that induced a stronger global lexical activity than non-derived pseudo-words, this depending on the higher number of orthographic neighbours. This hypothesis is supported by behavioural data showing faster RTs to letter strings than pseudo-words and to pseudo-words than quasi-words. This pattern of results agrees with the finding that reaction times to non-words are longer when these stimuli have many word neighbours [30].

### The timing of lexical processing

At posterior sites, over the left occipito/temporal area, the N3 response (345–395 ms) showed a gradient of activation with the highest response for the more familiar words and the lowest response for the less familiar word-like cluster of letters. This finding suggests an effect of visual familiarity of words as unitary visual objects. The relatively late onset of the lexical effect, compared to some recent literature [4,18,19,21,22], is very probably due to the mixed presentation of words and non-words with quasi-words that are very difficult to discriminate on the basis of visual appearance, since they were obtained by replacing just a single letter. In contrast, our data show that lexical effects may be very much delayed by the use of non-derived non-words with many orthographic neighbours [30,32,33]. In this regard, an important role in determining the onset of lexical effects is also played by the specific task modalities: for example, letter or phoneme detection (as in [20,50]) requiring focussed selective attention on the physical characteristics of the stimulus seems to expedite linguistic processing compared for example to a higher order task such as lexical decision, which was used in the present study and in others [34]. In addition, word length is a quite crucial factor in determining an earlier lexical onset for short (4–6 letters) vs. longer (7–9 letters) items [21].

Analysis of the anterior N2, LPN and P3 components suggests a dynamic analysis of word feature characteristics, which could be summarized as follows: at about 200–250 ms over the left fronto-central area, pseudo-words were discriminated from more word-like stimuli, resulting in a greater anterior negativity to pseudo-words as the earliest lexical effect. In the next latency range, at about 250–340 ms, the anterior frontal area showed a lexical gradient in the form of a lexical processing negativity that was very sensitive to word lexical properties and the number of orthographic neighbours. This effect might be conceived as a stage corresponding to the extraction (retrieval) of word semantic representations reflecting the global lexical activity of each item. At about 340–400 ms post-stimulus, the main stimulus property analyzed was word lexical representation: items lacking a sufficient level of lexical activation were therefore rejected as non-words. Indeed, P3 distinguished sharply between meaningful and meaningless stimuli, with no lexical gradient depending on well-formedness, legality or number of orthographic neighbours.

**Figure 10:**
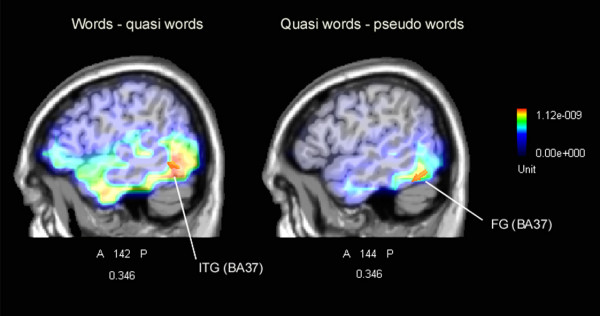
**(Left) Left sagittal view of swLORETA inverse solution performed on the difference wave obtained by subtracting ERPs to quasi-words from those elicited by words in the time window 345–395 ms.** Grid spacing = 5 mm; Tikhonov regularization: estimated SNR = 3; power RMS = 41.3 mV. The solution offered two strong sources of statistically significant activation explaining the surface difference-potential, one located in the left IT gyrus (BA 37), and the other in the right FG (BA37) of the temporal lobe. (**Right) **Right sagittal view of swLORETA inverse solution performed on the difference wave obtained by subtracting ERPs to quasi-words from those elicited by pseudo-words in the time window 345–395 ms. The linear inverse solution showed that the processing of more familiar non-words was significantly associated with stronger activity in the left FG (BA37) and in the right FG (BA37) of the temporal lobe. Power RMS = 27.7 mV.

The (late) lexical effects obtained in the present study were still earlier than those reported by Braun and colleagues [34]. These authors found a graded effect of non-word neighbours at about 500 ms post-stimulus, while the pure effect of lexicality was found at about 350 ms post-stimulus. This dissociation led the authors to interpret the data as reflecting two different decision processes: a faster identification process based on local lexical activity underlying the 'yes' response to words, and a slower temporal deadline process underlying the 'no' response to non-words based on global lexical activity. It should be considered that in their study the RTs were long, ranging from about 650 to 800 ms, whereas in the present experiment the response times did not exceed 620 ms. For this reason we found no time-delayed global lexical activity effects. On the contrary, the data suggest that orthographic, phonological and lexical word properties were processed in parallel between 200 and 400 ms post-stimulus. The first evidence that quasi-words benefited by their word-like visual form (thus leading to potentials of comparable amplitude between words and quasi-words) was observable at 200–250 ms at left front-central sites, while posteriorly, at about 350 ms, the left lateral-occipital region failed to discriminate them from words. In the same latency range, the nearby occipito/temporal area provided evidence of a marked discriminative response, with significantly enhanced amplitudes to words than quasi-words. In order to locate the possible neural source of this effect, a swLORETA source reconstruction was performed on the difference-wave obtained by subtracting ERPs to quasi-words from those elicited by words in the time window 345–395 ms (Figure 10, left). The linear inverse solution showed that the processing of real words was significantly associated with stronger activity in the left inferior temporal gyrus of the temporal lobe (X = -58.5, Y = -55.9, Z = -10.2, BA37) and in the right fusiform gyrus of the temporal lobe (X = 60.6, Y = -55, Z = -17.6, BA37). These data might be interpreted with the notion that, other things being equal (e.g. orthographic well-formedness), only real words possessing conceptual and sensory features might activate a region in the ventral stream that responds to complex objects and is crucial for recalling names of living entities (in this case, animals and vegetables) [51-53]. A further swLORETA aimed at assessing the possible neural locus of the visual word familiarity effect was performed on the difference-wave obtained by subtracting the ERPs to quasi-words from those elicited by pseudo-words in the time window 345–395 ms (Figure 10, right). The linear inverse solution showed that the processing of more familiar non-words (obtained by means of a single letter replacement) was significantly associated with stronger activity in the left fusiform gyrus of the temporal lobe (X = -48.5, Y = -55, Z = -17.6, BA37) and in the right fusiform gyrus of the temporal lobe (X = 50.8, Y = -55, Z = -17.6, BA37) (power RMS = 27.7 mV). This demonstrates that the occipito/temporal N350 might indicate the activity of the visual word form area (VWFA) devoted to orthographic processing, and sensitive to lexical or sub-lexical properties of words such as word familiarity [9,54-56].

A similarly late effect of word frequency on the occipito/temporal N2 and N3 components (240–360 ms), localized in the left fusiform gyrus of the occipital lobe, has been recently provided [46]. The data have been interpreted as an index of VWFA sub-lexical sensitivity. At this regard, it should be considered that a different degree of orthographic transparency (from the more transparent Italian to the deeper French or English orthographies) might play a role in the activation of a visual reading route.

## Conclusion

Overall, the data provided evidence that: (i) the latency of the lexical effect (word/non-word discrimination) varies as a function of the number of a word's orthographic neighbours, being faster to non-derived than to derived pseudo-words; this suggests some caveats in the use in lexical decision paradigms of quasi-words obtained by transposing or replacing only 1 or 2 letters. Our findings also showed that: (ii) the left-occipitotemporal area, probably reflecting the activity of the underlying VWFA (BA37), is sensitive to word visual familiarity, thus explaining its sub-lexical or even lexical sensitivity (word-pseudo-word difference); and (iii) phonological properties, accessed in a parallel modality during orthographic and lexical analysis, strongly affect lexical decision processes, allowing more rapid rejections of items lacking a phonological form.

## List of abbreviations

ANOVA: analysis of variance; BA: Brodmann area; EEG: electroencephalogram; EOG: electro-oculogram; ERPs: event-related potentials; LORETA: low resolution electromagnetic tomography; MEG: magnetoencephalography; RTs: reaction times; VWFA: visual word form area.

## Competing interests

The authors declare that they have no competing interests.

## Authors' contributions

AMP conceived of the study, coordinated data acquisition and analysis, interpreted the data and drafted the manuscript. RA participated in the design of the study, collected the data, performed statistical analyses and source localization.
